# Supplementary material for: Polyketide synthase gene clusters in the ericoid mycorrhizal fungus Oidiodendron maius: genome-wide prediction, expression profiling, and functional characterization of knockout mutants
Source: Front Plant Sci. 2026 Sep 16;17:1924430. doi: 10.3389/fpls.2026.1924430 (PMC13623903; doi:10.3389/fpls.2026.1924430)
Supplement: Supplementary file 1 [file SupplementaryFile1.zip › Supplementary Materials/Supplementary Folder 1.zip!/Oidma1_AssemblyScaffolds_Repeatmasked/knownclusterblast/cluster41/jgi.p_Oidma1_133417_mibig_hits.html]

| MIBiG Protein | Description | MIBiG Cluster | MiBiG Product | % ID | % Coverage | BLAST Score | E-value |
| --- | --- | --- | --- | --- | --- | --- | --- |
| ABA70582.1 | alpha-aminoadypil-cysteinyl-valine\_synthetase | BGC0000404 | Penicillin\_biosynthetic\_gene\_cluster | 46 | 101.063829787 | 578 | 1.1e-164 |
| ABR12615.1 | ACV\_synthetase | BGC0000405 | Penicillin\_biosynthetic\_gene\_cluster | 46 | 95.2887537994 | 544 | 2.3e-154 |
| KFH48607.1 | N-like\_protein | BGC0000317 | Cephalosporin\_biosynthetic\_gene\_cluster | 43 | 101.823708207 | 522 | 5.6e-148 |
| EDY47118.1 | N-(5-amino-5-carboxypentanoyl)-L-cysteinyl-D-\_valine\_synthase | BGC0000319 | Cephamycin\_C\_biosynthetic\_gene\_cluster | 36 | 101.975683891 | 406 | 5.8e-113 |
| CCA29203.1 | non-ribosomal\_peptide\_synthetase/polyketide\_synthase | BGC0000955 | Althiomycin\_biosynthetic\_gene\_cluster | 36 | 63.829787234 | 246 | 7.7e-65 |
| WP\_010369430.1 | Non-ribosomal\_peptide\_synthase | BGC0000314 | Bromoalterochromides\_biosynthetic\_gene\_cluster | 29 | 87.2340425532 | 196 | 1.2e-49 |
| WP\_010369428.1 | phenylalanine\_racemase | BGC0000314 | Bromoalterochromides\_biosynthetic\_gene\_cluster | 36 | 53.0395136778 | 191 | 3.8e-48 |
| ERG36783.1 | phenylalanine\_racemase | BGC0000299 | Alterochromides\_biosynthetic\_gene\_cluster | 36 | 53.0395136778 | 191 | 3.8e-48 |
| CBJ79916.1 | putative\_Ornithine\_racemase | BGC0001133 | Taxlllaid\_biosynthetic\_gene\_cluster | 31 | 66.717325228 | 191 | 3.8e-48 |
| CBJ79915.1 | putative\_Phenylalanine\_racemase\_(ATP-hydrolyzing) | BGC0001133 | Taxlllaid\_biosynthetic\_gene\_cluster | 31 | 66.1094224924 | 189 | 1.1e-47 |
| BAH43870.1 | putative\_linear\_pentadecapeptide\_gramicidin\_synthetase\_LgrB | BGC0000367 | Gramicidin\_biosynthetic\_gene\_cluster | 34 | 56.990881459 | 176 | 9.8e-44 |
| WP\_010369425.1 | ornithine\_racemase | BGC0000314 | Bromoalterochromides\_biosynthetic\_gene\_cluster | 30 | 64.7416413374 | 170 | 9.2e-42 |
| ERG36782.1 | Ornithine\_racemase | BGC0000299 | Alterochromides\_biosynthetic\_gene\_cluster | 30 | 64.7416413374 | 170 | 9.2e-42 |
| AAY93354.1 | non-ribosomal\_peptide\_synthetase\_PvdD | BGC0000413 | Pyoverdine\_biosynthetic\_gene\_cluster | 33 | 54.7112462006 | 169 | 1.2e-41 |
| BAH43871.1 | truncated\_linear\_pentadecapeptide\_gramicidin\_synthetase\_LgrC | BGC0000367 | Gramicidin\_biosynthetic\_gene\_cluster | 32 | 56.8389057751 | 166 | 1.3e-40 |
| AAY93445.1 | non-ribosomal\_peptide\_synthetase\_PvdL | BGC0000413 | Pyoverdine\_biosynthetic\_gene\_cluster | 31 | 67.1732522796 | 163 | 1.1e-39 |
| CAJ21198.2 | non-ribosomal\_peptide\_synthetase | BGC0000297 | Aeruginoside\_biosynthetic\_gene\_cluster | 29 | 68.5410334347 | 162 | 1.5e-39 |
| AHJ31215.1 | Long-chain-fatty-acid--CoA\_ligase | BGC0000430 | Spumigin\_biosynthetic\_gene\_cluster | 31 | 58.358662614 | 162 | 1.5e-39 |
| AFJ14794.1 | PlpE | BGC0000403 | Pelgipeptin\_biosynthetic\_gene\_cluster | 31 | 68.6930091185 | 160 | 9.5e-39 |
| AAO62586.1 | peptide\_sythetase | BGC0001016 | Microcystin\_biosynthetic\_gene\_cluster | 30 | 67.0212765957 | 158 | 2.1e-38 |
| CDG12864.1 | non-ribosomal\_peptide\_synthetase | BGC0001415 | Althiomycin\_biosynthetic\_gene\_cluster | 29 | 60.4863221884 | 158 | 3.6e-38 |
| BAB69700.1 | iturin\_A\_synthetase\_C | BGC0001098 | Iturin\_biosynthetic\_gene\_cluster | 30 | 69.3009118541 | 156 | 8e-38 |
| AIW82284.1 | PuwG | BGC0001125 | Puwainaphycins\_biosynthetic\_gene\_cluster | 33 | 57.5987841945 | 156 | 1.4e-37 |
| AEU11003.1 | NpnC | BGC0001029 | Nostophycin\_biosynthetic\_gene\_cluster | 34 | 52.1276595745 | 155 | 2.3e-37 |
| ANI24100.1 | nonribosomal\_peptide\_synthetase | BGC0001235 | Antalid\_biosynthetic\_gene\_cluster | 32 | 56.2310030395 | 155 | 3.1e-37 |
| AHF21228.1 | TriD | BGC0000449 | Tridecaptin\_biosynthetic\_gene\_cluster | 28 | 77.0516717325 | 153 | 1.2e-36 |
| ABS74179.1 | BmyC | BGC0001090 | Bacillomycin\_biosynthetic\_gene\_cluster | 28 | 68.9969604863 | 152 | 1.5e-36 |
| AGM16412.1 | paenibacterin\_synthetase\_A | BGC0000400 | Paenibacterin\_biosynthetic\_gene\_cluster | 29 | 75.2279635258 | 151 | 2.6e-36 |
| ACA97580.1 | PmxE | BGC0000408 | Polymyxin\_biosynthetic\_gene\_cluster | 28 | 79.9392097264 | 151 | 3.4e-36 |
| AAO23333.1 | NcpA | BGC0000397 | Nostocyclopeptide\_biosynthetic\_gene\_cluster | 29 | 71.7325227964 | 151 | 4.4e-36 |
| AGM16413.1 | paenibacterin\_synthetase\_B | BGC0000400 | Paenibacterin\_biosynthetic\_gene\_cluster | 32 | 56.990881459 | 150 | 5.8e-36 |
| AAY93356.2 | non-ribosomal\_peptide\_synthetase\_PvdI | BGC0000413 | Pyoverdine\_biosynthetic\_gene\_cluster | 31 | 56.3829787234 | 149 | 1.3e-35 |
| ABQ96384.2 | fusaricidin\_synthetase | BGC0001152 | Fusaricidin\_biosynthetic\_gene\_cluster | 29 | 68.5410334347 | 149 | 1.3e-35 |
| AEZ51520.1 | PmxE | BGC0001153 | Polymyxin\_biosynthetic\_gene\_cluster | 27 | 80.0911854103 | 149 | 1.7e-35 |
| AAU39359.1 | lichenysin\_synthase\_LchAA | BGC0000381 | Lichenysin\_biosynthetic\_gene\_cluster | 31 | 55.0151975684 | 147 | 6.4e-35 |
| AHZ20773.1 | non-ribosomal\_peptide\_synthase | BGC0000369 | Hassallidins\_biosynthetic\_gene\_cluster | 30 | 51.9756838906 | 147 | 6.4e-35 |
| AAC06346.1 | bacitracin\_synthetase\_1 | BGC0000310 | Bacitracin\_biosynthetic\_gene\_cluster | 29 | 66.5653495441 | 146 | 1.1e-34 |
| ADJ63842.1 | non-ribosomal\_peptide\_synthetase\_protein | BGC0000424 | Serobactins\_biosynthetic\_gene\_cluster | 30 | 56.990881459 | 146 | 1.4e-34 |
| CAJ46692.1 | non\_ribosomal\_peptide\_synthase | BGC0000969 | Chondramide\_biosynthetic\_gene\_cluster | 29 | 68.6930091185 | 145 | 1.9e-34 |
| AJM89738.1 | PmxE | BGC0001192 | Colistin\_biosynthetic\_gene\_cluster | 28 | 80.2431610942 | 145 | 1.9e-34 |
| AHD05678.1 | nonribosomal\_peptide\_ligase\_subunit | BGC0000402 | Paenilarvins\_biosynthetic\_gene\_cluster | 33 | 56.3829787234 | 145 | 2.4e-34 |
| ACZ55942.1 | non-ribosomal\_peptide\_synthetase | BGC0000302 | Anabaenopeptin\_biosynthetic\_gene\_cluster | 30 | 56.5349544073 | 144 | 3.2e-34 |
| ACA09733.2 | fusaricidin\_synthetase | BGC0000358 | Fusaricidin\_biosynthetic\_gene\_cluster | 29 | 68.5410334347 | 144 | 3.2e-34 |
| AAF08797.1 | MycC | BGC0001103 | Mycosubtilin\_biosynthetic\_gene\_cluster | 29 | 69.452887538 | 144 | 4.1e-34 |
| CAD29797.1 | peptide\_synthetase | BGC0001015 | Microcystin\_biosynthetic\_gene\_cluster | 28 | 68.2370820669 | 144 | 4.1e-34 |
| CAE02630.1 | surfactin\_synthetase\_A | BGC0000433 | Surfactin\_biosynthetic\_gene\_cluster | 30 | 63.2218844985 | 144 | 4.1e-34 |
| ABS74209.1 | FenA | BGC0001095 | Fengycin\_biosynthetic\_gene\_cluster | 32 | 55.7750759878 | 144 | 5.4e-34 |
| CAE02631.1 | surfactin\_synthetase\_B | BGC0000433 | Surfactin\_biosynthetic\_gene\_cluster | 28 | 68.9969604863 | 144 | 5.4e-34 |
| AJM89735.1 | PmxA | BGC0001192 | Colistin\_biosynthetic\_gene\_cluster | 27 | 69.452887538 | 142 | 2e-33 |
| ACZ55945.1 | non-ribosomal\_peptide\_synthetase | BGC0000302 | Anabaenopeptin\_biosynthetic\_gene\_cluster | 30 | 56.990881459 | 141 | 2.7e-33 |
| AEW31021.1 | plipastatin\_synthetase | BGC0000407 | Plipastatin\_biosynthetic\_gene\_cluster | 27 | 70.9726443769 | 141 | 4.6e-33 |
| AHF21229.1 | TriE | BGC0000449 | Tridecaptin\_biosynthetic\_gene\_cluster | 32 | 52.5835866261 | 141 | 4.6e-33 |
| ABD14711.1 | CesA | BGC0000320 | Cereulide\_biosynthetic\_gene\_cluster | 25 | 73.556231003 | 140 | 6e-33 |
| AHZ20774.1 | non-ribosomal\_peptide\_synthase | BGC0000369 | Hassallidins\_biosynthetic\_gene\_cluster | 31 | 57.5987841945 | 140 | 7.8e-33 |
| AEU11006.1 | NpnB | BGC0001029 | Nostophycin\_biosynthetic\_gene\_cluster | 32 | 53.4954407295 | 139 | 1e-32 |
| AEW31019.1 | plipastatin\_synthetase | BGC0000407 | Plipastatin\_biosynthetic\_gene\_cluster | 32 | 57.9027355623 | 139 | 1.3e-32 |
| AAU39360.1 | lichenysin\_synthase\_LchAB | BGC0000381 | Lichenysin\_biosynthetic\_gene\_cluster | 29 | 55.623100304 | 138 | 2.3e-32 |
| AHI59109.1 | locillomycin\_synthase\_B | BGC0001005 | Locillomycin\_biosynthetic\_gene\_cluster | 29 | 55.7750759878 | 138 | 2.3e-32 |
| BAB69699.1 | iturin\_A\_synthetase\_B | BGC0001098 | Iturin\_biosynthetic\_gene\_cluster | 31 | 55.1671732523 | 138 | 2.3e-32 |
| AAC06348.1 | bacitracin\_synthetase\_3 | BGC0000310 | Bacitracin\_biosynthetic\_gene\_cluster | 27 | 69.7568389058 | 138 | 3e-32 |
| AHD05677.1 | nonribosomal\_peptide\_ligase\_subunit | BGC0000402 | Paenilarvins\_biosynthetic\_gene\_cluster | 26 | 68.9969604863 | 138 | 3.9e-32 |
| AEZ51516.1 | PmxA | BGC0001153 | Polymyxin\_biosynthetic\_gene\_cluster | 27 | 69.452887538 | 137 | 5e-32 |
| AAC06347.1 | bacitracin\_synthetase\_2 | BGC0000310 | Bacitracin\_biosynthetic\_gene\_cluster | 27 | 68.8449848024 | 135 | 2.5e-31 |
| AHD05617.1 | putative\_non-ribosomal\_peptide\_ligase\_domain\_protein | BGC0001033 | Paenilamicin\_biosynthetic\_gene\_cluster | 28 | 66.8693009119 | 134 | 3.3e-31 |
| AEA30274.1 | peptide\_synthetase | BGC0000429 | Skyllamycin\_biosynthetic\_gene\_cluster | 29 | 58.0547112462 | 134 | 3.3e-31 |
| ABS74180.1 | BmyB | BGC0001090 | Bacillomycin\_biosynthetic\_gene\_cluster | 31 | 55.3191489362 | 134 | 4.3e-31 |
| BAH43765.1 | tyrocidine\_synthetase\_II | BGC0000452 | Tyrocidine\_biosynthetic\_gene\_cluster | 27 | 67.6291793313 | 133 | 7.3e-31 |
| CAQ71829.1 | non\_ribosomal\_peptide\_synthase,\_antibiotic\_synthesis;\_contains\_4\_condensation\_domains,\_3\_AMP-acid\_ligases\_II\_domains,\_3\_PP-binding,\_Phosphopantetheine\_attachment\_site\_and\_a\_putative\_thioesterase\_domain | BGC0001189 | Taiwachelin\_biosynthetic\_gene\_cluster | 29 | 66.717325228 | 133 | 9.5e-31 |
| CAQ71828.1 | non\_ribosomal\_peptide\_synthase,\_antibiotic\_synthesis;\_contains\_3\_condensation\_domains,\_2\_AMP-acid\_ligases\_II\_domains,\_2\_PP-binding,\_Phosphopantetheine\_attachment\_site | BGC0001189 | Taiwachelin\_biosynthetic\_gene\_cluster | 30 | 60.3343465046 | 132 | 2.1e-30 |
| ACA97576.1 | PmxA | BGC0000408 | Polymyxin\_biosynthetic\_gene\_cluster | 26 | 69.452887538 | 131 | 2.8e-30 |
| CAJ96470.1 | Non-ribosomal\_peptide\_synthetase | BGC0000330 | Cupriachelin\_biosynthetic\_gene\_cluster | 28 | 57.2948328267 | 131 | 3.6e-30 |
| AGZ03650.1 | SevA | BGC0000426 | Sevadicin\_biosynthetic\_gene\_cluster | 30 | 56.8389057751 | 131 | 4.7e-30 |
| AAF08796.1 | MycB | BGC0001103 | Mycosubtilin\_biosynthetic\_gene\_cluster | 28 | 66.717325228 | 129 | 1.4e-29 |
| BAH43869.1 | linear\_pentadecapeptide\_gramicidin\_synthetase\_LgrA | BGC0000367 | Gramicidin\_biosynthetic\_gene\_cluster | 26 | 67.6291793313 | 128 | 3.1e-29 |
| AHD05627.1 | putative\_non-ribosomal\_peptide\_ligase\_domain\_protein | BGC0001033 | Paenilamicin\_biosynthetic\_gene\_cluster | 27 | 70.3647416413 | 127 | 6.8e-29 |
| ACM79810.1 | ZmaO | BGC0001059 | Zwittermycin\_A\_biosynthetic\_gene\_cluster | 26 | 65.5015197568 | 124 | 3.4e-28 |
| AEC14346.1 | nonribosomal\_peptide\_synthetase | BGC0000377 | Koranimine\_biosynthetic\_gene\_cluster | 29 | 53.9513677812 | 123 | 9.8e-28 |
| ABX37382.1 | amino\_acid\_adenylation\_domain\_protein | BGC0000984 | Delftibactin\_biosynthetic\_gene\_cluster | 31 | 50.0 | 123 | 9.8e-28 |
| AEC14347.1 | nonribosomal\_peptide\_synthetase | BGC0000377 | Koranimine\_biosynthetic\_gene\_cluster | 24 | 68.9969604863 | 123 | 9.8e-28 |
| AAU34203.1 | mannopeptimycin\_peptide\_synthetase\_MppB | BGC0000388 | Mannopeptimycin\_biosynthetic\_gene\_cluster | 29 | 56.3829787234 | 121 | 2.9e-27 |
| AEC14348.1 | nonribosomal\_peptide\_synthetase | BGC0000377 | Koranimine\_biosynthetic\_gene\_cluster | 24 | 68.5410334347 | 121 | 2.9e-27 |
| AEP18655.1 | WAPS2 | BGC0000461 | WAP-8294A2\_(lotilibcin)\_biosynthetic\_gene\_cluster | 27 | 64.5896656535 | 121 | 3.7e-27 |
| EWS95122.1 | hypothetical\_protein | BGC0000306 | Arylomycin\_biosynthetic\_gene\_cluster | 29 | 53.3434650456 | 120 | 6.4e-27 |
| AEW31022.1 | plipastatin\_synthetase | BGC0000407 | Plipastatin\_biosynthetic\_gene\_cluster | 25 | 73.2522796353 | 119 | 1.4e-26 |
| AJV88375.1 | MfnC | BGC0001214 | Marformycins\_biosynthetic\_gene\_cluster | 29 | 64.5896656535 | 119 | 1.4e-26 |
| AEH41793.1 | HrmO | BGC0000374 | Hormaomycin\_biosynthetic\_gene\_cluster | 28 | 58.9665653495 | 119 | 1.9e-26 |
| ALV82384.1 | CDA\_peptide\_synthetase\_II | BGC0001370 | Lipopeptide\_8D1-1\_/\_lipopeptide\_8D1-2\_biosynthetic\_gene\_cluster | 26 | 59.4224924012 | 113 | 1e-24 |
| AAC32048.1 | FxbC | BGC0000351 | Exochelin\_biosynthetic\_gene\_cluster | 28 | 49.6960486322 | 113 | 1e-24 |
| AJW76710.1 | DsaH | BGC0001196 | Desotamide\_biosynthetic\_gene\_cluster | 27 | 59.4224924012 | 112 | 1.7e-24 |
| AEP18656.1 | WAPS1 | BGC0000461 | WAP-8294A2\_(lotilibcin)\_biosynthetic\_gene\_cluster | 29 | 57.5987841945 | 109 | 1.5e-23 |
| AEG64696.1 | LpmB | BGC0000379 | Laspartomycin\_biosynthetic\_gene\_cluster | 27 | 63.0699088146 | 109 | 1.9e-23 |
| ALK27915.1 | non-ribosomal\_peptide\_synthase | BGC0001233 | Feglymycin\_biosynthetic\_gene\_cluster | 28 | 54.5592705167 | 108 | 2.5e-23 |
| sln7 | nonribosomal\_peptide\_synthetase | BGC0001230 | Salinamides\_biosynthetic\_gene\_cluster | 27 | 67.0212765957 | 108 | 4.3e-23 |
| CAM02313.1 | putative\_non-ribosomal\_peptide\_synthetase | BGC0000349 | Erythrochelin\_biosynthetic\_gene\_cluster | 28 | 62.0060790274 | 107 | 5.6e-23 |
| AFK57215.1 | DidD | BGC0000985 | Didemnin\_biosynthetic\_gene\_cluster | 25 | 67.1732522796 | 106 | 9.5e-23 |
| sln8 | nonribosomal\_peptide\_synthetase | BGC0001230 | Salinamides\_biosynthetic\_gene\_cluster | 27 | 60.7902735562 | 106 | 1.2e-22 |
| AMK48226.1 | nonribosomal\_peptide\_synthetase | BGC0001351 | Cahuitamycins\_biosynthetic\_gene\_cluster | 25 | 66.717325228 | 105 | 2.1e-22 |
| AIG79241.1 | Hypothetical\_protein | BGC0000419 | Ristomycin\_A\_biosynthetic\_gene\_cluster | 27 | 68.3890577508 | 105 | 2.8e-22 |
| ADY16697.1 | TqaA | BGC0001142 | Tryptoquialanine\_biosynthetic\_gene\_cluster | 29 | 51.367781155 | 103 | 8.1e-22 |
| AEG64697.1 | LpmC | BGC0000379 | Laspartomycin\_biosynthetic\_gene\_cluster | 26 | 57.7507598784 | 102 | 1.4e-21 |
| ALG65318.1 | Cal18 | BGC0001297 | WS9326\_biosynthetic\_gene\_cluster | 24 | 61.7021276596 | 100 | 6.8e-21 |
| AIE77059.1 | peptide\_synthetase | BGC0000418 | Ristocetin\_biosynthetic\_gene\_cluster | 26 | 57.9027355623 | 100 | 8.9e-21 |
| AID65222.1 | putative\_aspartate\_racemase | BGC0000335 | Cystomanamides\_biosynthetic\_gene\_cluster | 26 | 55.9270516717 | 99 | 1.5e-20 |
| BAE98155.1 | putative\_non-ribosomal\_peptide\_synthetase | BGC0000339 | Echinomycin\_biosynthetic\_gene\_cluster | 27 | 58.358662614 | 98 | 2.6e-20 |
| AAZ23077.1 | peptide\_synthetase | BGC0000291 | A54145\_biosynthetic\_gene\_cluster | 26 | 69.7568389058 | 98 | 2.6e-20 |
| AJV88376.1 | MfnD | BGC0001214 | Marformycins\_biosynthetic\_gene\_cluster | 25 | 59.726443769 | 98 | 3.4e-20 |
| CAD91212.1 | putative\_non-ribosomal\_peptide\_synthetase,\_modules\_4-6 | BGC0000289 | A40926\_biosynthetic\_gene\_cluster | 26 | 60.3343465046 | 98 | 3.4e-20 |
| CAJ18237.2 | non-ribosomal\_peptide\_synthetase\_B | BGC0000354 | Friulimicin\_biosynthetic\_gene\_cluster | 24 | 60.3343465046 | 97 | 4.4e-20 |
| WP\_020635019.1 | hypothetical\_protein | BGC0001211 | Albachelin\_biosynthetic\_gene\_cluster | 27 | 58.358662614 | 96 | 9.9e-20 |
| CAJ34374.1 | NRPS\_protein | BGC0000445 | Thiocoraline\_biosynthetic\_gene\_cluster | 26 | 57.7507598784 | 94 | 4.9e-19 |
| AEI58866.1 | peptide\_synthetase | BGC0000455 | Vancomycin\_biosynthetic\_gene\_cluster | 27 | 56.2310030395 | 94 | 4.9e-19 |
| EAL89049.1 | nonribosomal\_peptide\_synthase,\_putative | BGC0000355 | Fumiquinazolines\_biosynthetic\_gene\_cluster | 27 | 54.4072948328 | 92 | 1.4e-18 |
| CAC48361.1 | peptide\_synthetase | BGC0000311 | Balhimycin\_biosynthetic\_gene\_cluster | 25 | 60.9422492401 | 91 | 4.1e-18 |
| AAT01806.1 | non-ribosomal\_peptide\_synthetase | BGC0000365 | Glycopeptidolipid\_biosynthetic\_gene\_cluster | 26 | 68.3890577508 | 91 | 5.4e-18 |
| ABV56588.1 | KtzH | BGC0000378 | Kutznerides\_biosynthetic\_gene\_cluster | 26 | 63.3738601824 | 90 | 7.1e-18 |
| CBZ42146.1 | putative\_non-ribosomal\_peptide\_synthetase | BGC0001117 | Himastatin\_biosynthetic\_gene\_cluster | 27 | 53.7993920973 | 89 | 1.6e-17 |
| AHH53507.1 | non-ribosomal\_peptide\_synthetase | BGC0000439 | Taromycin\_biosynthetic\_gene\_cluster | 34 | 37.537993921 | 89 | 1.6e-17 |
| CAJ77696.1 | MPS2\_protein | BGC0000363 | Glycopeptidolipid\_biosynthetic\_gene\_cluster | 31 | 40.8814589666 | 89 | 1.6e-17 |
| CAA11795.1 | PCZA363.4 | BGC0000322 | Chloroeremomycin\_biosynthetic\_gene\_cluster | 25 | 61.094224924 | 88 | 2.7e-17 |
| BAO73252.1 | non-ribosomal\_peptide\_synthetase | BGC0000456 | Verlamelin\_biosynthetic\_gene\_cluster | 28 | 47.8723404255 | 88 | 3.5e-17 |
| AGZ15459.1 | putative\_non-ribosomal\_peptide\_synthetase | BGC0001036 | Polyoxypeptin\_biosynthetic\_gene\_cluster | 31 | 37.6899696049 | 87 | 4.6e-17 |
| CCB53264.1 | non-ribosomal\_peptide\_synthetase | BGC0001393 | Lugdunin\_biosynthetic\_gene\_cluster | 27 | 48.1762917933 | 87 | 4.6e-17 |
| CAJ77715.1 | Mps1\_protein | BGC0000364 | Glycopeptidolipid\_biosynthetic\_gene\_cluster | 25 | 59.4224924012 | 87 | 6e-17 |
| CAJ77716.1 | Mps2\_protein | BGC0000364 | Glycopeptidolipid\_biosynthetic\_gene\_cluster | 32 | 39.0577507599 | 85 | 3e-16 |
| EFL06865.1 | hypothetical\_protein | BGC0000300 | Amychelin\_biosynthetic\_gene\_cluster | 28 | 45.896656535 | 84 | 6.6e-16 |
| CAJ77695.1 | MPS1\_protein | BGC0000363 | Glycopeptidolipid\_biosynthetic\_gene\_cluster | 25 | 57.2948328267 | 84 | 6.6e-16 |
| ctg1\_orf000000 |  | BGC0000901 | Ferrichrome\_biosynthetic\_gene\_cluster | 24 | 70.5167173252 | 83 | 1.1e-15 |
| ACU36654.1 | amino\_acid\_adenylation\_domain\_protein | BGC0000392 | Mirubactin\_biosynthetic\_gene\_cluster | 24 | 53.7993920973 | 83 | 1.1e-15 |
| AAD44233.1 | PstA | BGC0000362 | Glycopeptidolipid\_biosynthetic\_gene\_cluster | 24 | 58.5106382979 | 82 | 1.9e-15 |
| AAT01807.1 | non-ribosomal\_peptide\_synthetase | BGC0000365 | Glycopeptidolipid\_biosynthetic\_gene\_cluster | 30 | 40.4255319149 | 81 | 3.3e-15 |
| BAH04162.1 | trsJ | BGC0000450 | Triostin\_A\_biosynthetic\_gene\_cluster | 24 | 48.1762917933 | 81 | 4.3e-15 |
| AAZ23075.1 | peptide\_synthetase | BGC0000291 | A54145\_biosynthetic\_gene\_cluster | 24 | 60.6382978723 | 81 | 4.3e-15 |
| AHH53506.1 | non-ribosomal\_peptide\_synthetase | BGC0000439 | Taromycin\_biosynthetic\_gene\_cluster | 24 | 57.4468085106 | 80 | 7.3e-15 |
| CBW45640.1 | PII\_peptide\_synthetase | BGC0000952 | Pristinamycin\_biosynthetic\_gene\_cluster | 23 | 55.4711246201 | 74 | 5.2e-13 |
| EFY94500.1 | non-ribosomal\_peptide\_synthetase | BGC0000337 | Destruxins\_biosynthetic\_gene\_cluster | 25 | 48.7841945289 | 74 | 5.2e-13 |
| AAD44234.1 | PstB | BGC0000362 | Glycopeptidolipid\_biosynthetic\_gene\_cluster | 23 | 55.7750759878 | 71 | 4.4e-12 |
| WP\_028678149.1 | non-ribosomal\_peptide\_synthetase | BGC0001228 | Retimycin\_biosynthetic\_gene\_cluster | 27 | 38.1458966565 | 70 | 7.6e-12 |
| CAN89661.1 | putative\_non-ribosomal\_peptide\_synthetase | BGC0001070 | Kirromycin\_biosynthetic\_gene\_cluster | 29 | 40.5775075988 | 68 | 2.9e-11 |
| CAP93139.1 |  | BGC0000357 | Fungisporin\_biosynthetic\_gene\_cluster | 25 | 52.1276595745 | 65 | 2.4e-10 |
| AJI44167.1 | long-chain-fatty-acid-CoA\_ligase | BGC0001193 | Thiolutin\_biosynthetic\_gene\_cluster | 25 | 31.7629179331 | 63 | 1.2e-09 |
| CBF87069.1 | nonribosomal\_peptide\_synthase,\_putative\_(Eurofung) | BGC0001290 | Emericellamide\_A\_/\_emericellamide\_B\_biosynthetic\_gene\_cluster | 23 | 49.6960486322 | 63 | 1.2e-09 |
| AEA30272.1 | peptide\_synthetase | BGC0000429 | Skyllamycin\_biosynthetic\_gene\_cluster | 24 | 60.4863221884 | 57 | 5.1e-08 |
| AAN32981.1 | BarG | BGC0000962 | Barbamide\_biosynthetic\_gene\_cluster | 28 | 24.6200607903 | 54 | 5.6e-07 |
| BAH43766.1 | tyrocidine\_synthetase\_III | BGC0000452 | Tyrocidine\_biosynthetic\_gene\_cluster | 23 | 43.7689969605 | 52 | 2.1e-06 |
